# Pathologically Relevant Mouse Models for Epstein–Barr Virus–Associated B Cell Lymphoma

**DOI:** 10.3389/fimmu.2021.639844

**Published:** 2021-02-24

**Authors:** Shiyu Huang, Tomoharu Yasuda

**Affiliations:** Department of Immunology, Graduate School of Biomedical and Health Sciences, Hiroshima University, Hiroshima, Japan

**Keywords:** Epstein-Barr virus, B cell lymphoma, immune surveillance, mouse model, lymphoproliferative disease

## Abstract

The Epstein–Barr virus (EBV) is endemic in humans and can efficiently transform infected B cells under some circumstances. If an EBV carrier experiences immune suppression, EBV^+^ B cells can turn into lymphoblasts and exhibit growth expansion that may cause lymphoproliferative diseases which often develop into lymphoma. Our immune system conducts surveillance for EBV^+^ B cells in order to block spontaneous tumor formation. Here, we summarize the EBV products involved in tumorigenesis, EBV-associated lymphomas, and pathologically relevant mouse models. Preclinical mouse models for a range of EBV-associated diseases not only clear the path to new therapeutic approaches but also aid in our understanding of the nature of lymphomagenesis and immune surveillance.

## Introduction

Epstein–Barr virus (EBV), an oncogenic γ herpes virus, is widespread in all human populations and persists in the vast majority of individuals throughout their lifetime. EBV preferentially infects B cells through the CD21/CR2 receptor on the surface of B cells, which is an entry receptor for viral envelope glycoprotein gp350. Viral glycoprotein gp42 interacts with cellular human leukocyte antigen (HLA) Class II molecules as a co-receptor, triggering fusion of the viral envelope with the cell membrane (1). Primary infection in young children is usually asymptomatic, but if infection is delayed until adolescence, it can cause infectious mononucleosis (IM) accompanied by EBV-infected B cell expansion and enormous T cell activation (2). After the acute phase of infection, EBV persists in a small subset of memory B cells (0.0001–0.005% of peripheral blood B cells) throughout the patient's lifetime, and is maintained as silent because of the specifically established memory T cells (3–5). Although EBV is usually a harmless passenger, immunocompromised individuals can develop severe complications. Genetic defects that lead to impaired T cell function predispose individuals to EBV-driven lymphoproliferative diseases or hematological diseases such as X-linked lymphoproliferative disease (XLP) or familial hemophagocytic lymphohistiocytosis (FHL) (6, 7). Lymphomas associated with post-transplant lymphoproliferative diseases (PTLDs) arising in patients receiving immunosuppressive drug treatment after organ transplantation are usually positive for EBV. Human immunodeficiency virus (HIV) infection can also lead to the development of EBV^+^ lymphoma called acquired immunodeficiency syndrome (AIDS)-related lymphoma. Thus, in immune suppression, B cells carrying the EBV episome turn into activated lymphoblasts, which later often develop into lymphoma. Furthermore, several escape mechanisms from EBV-specific immunity lead to EBV^+^ lymphoma even in immunocompetent people, including Burkitt lymphoma (BL), Hodgkin's lymphoma (HL), and non-Hodgkin's lymphoma (NHL). EBV may also act as a passenger in cases in which malignant transformation occurs in an EBV-infected B-lymphocyte. Although many B cell malignancies are associated with EBV infection, the precise roles of EBV in the tumorigenic process and immune escape remain largely unknown. Therefore, the development of preclinical mouse models for EBV infection and the pathogenesis of EBV-associated lymphoma is important as it could open up new therapeutic modalities.

## EBV Products in Latently Infected B Cells

Upon EBV infection, each infected cell carries multiple extrachromosomal copies of viral episomes and constitutively expresses a limited set of viral gene products called latent proteins. Among EBV-encoded genes, nine viral proteins can be expressed from latently infected EBV episomes to maintain the viral genome and regulate host B cell properties: EBV-nuclear antigens EBNA1, EBNA2, EBNA3A, EBNA3B, EBNA3C, EBNA-LP, and latent membrane proteins LMP1, LMP2A, and LMP2B. Additionally, it also expresses non-coding RNAs EBER1, EBER2, BART miRNAs, and BHRF-1 miRNAs. EBV exhibits one of four latency programs, latency 0, I, II, or III, depending on the status of B cells and lymphoma types (Table 1). EBV can persist in resting B cells without expressing viral genes escaping from the immune system for a long period (latency 0) or they can express either latency I, II, or III depending on the disease type (8).

**Table 1:** EBV latency programs and EBV-associated lymphomas.

**Latent programs**	**Latency I**	**Latency II**	**Latency III**
Expressed EBV non-coding RNAs	EBER1/2, BART miRNAs	EBER1/2, BART miRNAs	EBER1/2, BART miRNAs
Expressed EBV mRNAs	EBNA1	EBNA1	EBNA1
		LMP1, LMP2A, LMP2B	LMP1, LMP2A, LMP2B
			EBNA2, EBNA3A EBNA3B, EBNA3C EBNA-LP
Sensitivity to CTL	Resistant	Resistant ~ Sensitive	Sensitive
EBV-associated lymphomas (Association, %)	BL, endemic (95–100) BL, sporadic (20–30) AIDS-BL (55) AIDS-PEL (90–100) AIDS-DLBCL-CB (30)	HL, Western world (40) HL, Children in Central America (90) AIDS-HL (100) DLBCL (10–15)	PTLD (80) AIDS-PCNSL (100) AIDS-DLBCL-IB (90)

*AIDS, Acquired immunodeficiency syndrome; BART, BamHI-A rightward reading frame transcript; BL, Burkitt lymphoma; CB, Centroblast; CTL, Cytotoxic T-lymphocyte; DLBCL, Diffuse large B-cell lymphoma; EBERs, EBV-encoded small RNAs; EBNA, EBV nuclear antigen; HL, Hodgkin's lymphoma; IB, Immunoblast; LMP, Latent membrane protein; PCNSL, Primary central nervous system lymphoma; PEL, Primary effusion lymphoma; PTLD, Post-transplant lymphoproliferative disease*.

## Latent Membrane Proteins (LMPs)

LMP1 is an important oncogene encoded by EBV that is expressed in many types of EBV-associated lymphomas (Table 1). LMP1 containing intracellular signals and transmembrane domains can promote self-aggregation in the plasma membrane that transmits constitutive intracellular signal mimicking CD40, an important costimulatory molecule for B cells. LMP1 mimics an active CD40 receptor and recruits tumor necrosis factor (TNF) receptor–associated factor (TRAF) in the COOH terminal cytoplasmic region. LMP1 expression induces NF-κB and MAPK activation, and upregulates anti-apoptotic factors A20, Bcl-2, and proto-oncogene c-Myc. The expression of LMP1 in B cells induces blastic change and massive proliferation, which eventually transform B cells. EBV lacking LMP1 is unable to transform B cells (9, 10). It is of note that LMP1 expression is known to be heterogenous in B-NHL. A meta-analysis demonstrated that LMP1 expression is an unfavorable prognostic factor for overall survival in NHL patients (11). LMP2A also contains signaling and transmembrane domains that transmit constitutive signals mimicking the B cell receptor (BCR). LMP2A enhances the expression of genes related to cell cycle induction and apoptosis inhibition, and changes the expression of genes related to cell metabolism. LMP2A transmits signals through Lyn and Syk, which can replace BCR in B cell development (12, 13). EBV-associated lymphomas originate from germinal center (GC) B cells. Although the BCR signal plays an important role in GC-derived lymphoma cells, GC B cells characteristically downregulate BCR expression and its signal, suggesting that EBV may replace BCR function. In addition, lymphoma derived from GC B cells such as HLs often lose BCR expression because of deleterious somatic mutations in their immunoglobulin genes. Importantly, the EBV-mediated transformation of both BCR^+^ and BCR^−^ GC B cells is strictly dependent on LMP2A expression (14).

## EBV Nuclear Antigens and Non-coding RNA

EBNA1 is a DNA-binding nuclear phospho-protein that plays a central role in the replication and maintenance of the episomal EBV genome. Directing EBNA1 expression to B cells in transgenic mice results in B cell lymphomas, suggesting that EBNA1 might have a direct role in oncogenesis, although there is no evidence explaining the direct role of EBNA1 in the immortalization or transformation of B cells (15). EBNA2 is expressed early after infection and has an important role in the immortalization of B cells through the induction of viral genes such as LMP1 and LMP2A. EBNA2, which mimics Notch in binding to RBP-Jκ and activating cellular target genes (most notably Myc), is an essential molecule in human B cell growth transformation by EBV (8, 16). A recent study identified EBNA2 as a lead player in tampering with the immunogenicity of EBV^+^ B cell lymphoma by altering PD-L1 expression (17). The EBNA3 family are transcriptional regulators controlling RBP-Jκ activity, and thereby regulate the activity of cellular and viral promoters. Studies with EBV recombinants have shown that EBNA3A and EBNA3C are essential for human B cell transformation *in vitro* whereas EBNA3B is non-essential (18). Recently, EBNA3A was shown to promote LMP1- and LMP2A-induced lymphomagenesis in mice by inhibiting the terminal differentiation of lymphoma progenitors and cooperating with c-Myc expression (19). EBNA-LP is not required for human B cell transformation *in vitro*, but is required for the efficient outgrowth of LCLs (20). EBNA-LP promotes the cell cycle through the induction of Cyclin D2 together with EBNA2 (21). The ability of EBNA-LP to enhance EBNA2-mediated transactivation suggests its importance in EBV-driven lymphomagenesis. In addition to the latent proteins, non-coding RNA, EBER1, and EBER2 are consistently expressed in all forms of latent EBV infection (Table 1). EBER blocks IFN-induced apoptosis by binding to dsRNA-activated protein kinase (PKR). The role of EBER in IL-10 production in BL cells has been shown. EBER promotes the induction of autocrine growth factors IL-10, IL-9, and insulin-like growth factor-1 (22). The transgenic expression of EBER1 in the mouse B cell compartment promotes hyperplasia and Myc-induced lymphoma development (23). Therefore, EBERs may contribute to tumor growth or escape from the immune system. BART miRNAs (encoding 44 mature BART miRNAs) are expressed in all latency types whereas BHRF1 miRNAs (encoding 4 mature BHRF1 miRNAs) are only expressed in type III latency. Although the aberrant expression of these miRNAs may be involved in transformation and tumor growth, the precise role has not yet been established (24).

## Burkitt Lymphoma

BL was the first B cell lymphoma discovered to be associated with EBV. BL is characterized by c-Myc chromosomal translocation and is subdivided into three types, endemic, sporadic, and immunodeficiency, all of which are associated with EBV. Endemic and sporadic BL account for 95–100 and 20–30% of EBV-associated lymphomas, respectively (Table 1). Nearly all BL carries c-Myc/Ig translocation *t*_(8;14)_, *t*_(2;8)_, or *t*_(8;22)_ leading to dysregulated c-Myc proto-oncogene expression, indicating the critical role of c-Myc in Burkitt lymphomagenesis (25). In EBV-positive BLs, only EBNA1 is expressed besides EBERs and BART miRNAs (latency I). Other EBV latent genes are downregulated, and lymphoma cells are thus hidden from EBV-specific host memory T cells. Functional inhibition of EBNA1 eradicates the EBV episome and prevents the malignant phenotype of EBV^+^ BL cells (26, 27). The constitutive expression of c-Myc together with the active form of phosphoinositide-3-kinase (PI3K) in germinal center B cells, an origin of BL, synergistically induces mouse B cell lymphoma similar to human BL (28). This study suggests that the anti-apoptotic survival signal coinciding with constitutive c-Myc expression is sufficient and critical to the development of BL. Transgenic expression of EBER1 in B cells (Eμ-EBER1) together with c-Myc- or N-Myc promoted Myc-dependent lymphomagenesis, suggesting the role of EBER1 in lymphomagenesis (23). Because the synergistic effect of Myc and EBER1 was relatively mild compared to Myc and active PI3K, however, additional events promoting cell survival might be required for BL tumor formation. Indeed, oncogenic mutations in the pro-survival genes, cell cycle regulator, and transcriptional regulators were found (29), and these additional mutations might be required for the lymphomagenesis from Myc-translocated EBV^+^ GC B cells.

## Hodgkin's Lymphoma

HL is characterized by atypical, large tumor cells known as Hodgkin and Reed–Sternberg (HRS) cells. These cells usually represent <1% of the tumor tissue; most tumor cells are non-malignant T cells and other immune cells (8). In the Western world, EBV is detected in ~40% of HRS cells in classical HL. In Latin America, nearly all cases of HL in children are EBV-positive. In EBV-positive cases, three EBV proteins, EBNA1, LMP1, and LMP2A, are expressed (latency II). HRS cells are equipped with mechanisms to escape immune surveillance, including the downregulation of MHC class I and II, and the overexpression of CTL suppressor molecules, such as PD-L1, PD-L2, TGF-β, IL-10, Gal-1, Fas-L, and Treg-attracting chemokines (30). HRS cells originate from GC B cells. The rearranged Ig V genes of HRS are somatically mutated but lack intra-clonal diversity, indicating that the SHM machinery is silenced in tumor cells. Interestingly, 25% of HRS cells carry non-sense or deleterious mutations in the functional immunoglobulin gene. GC B cells that acquire such crippling mutations are normally eliminated by apoptosis in the GC reaction, but the expression of LMP2A by EBV infection might allow the survival of GC B cells with crippling mutations as LMP2A could replace BCR (12, 13). To date, no mouse models relevant to HL disease have been successfully established.

## Diffuse Large B Cell Lymphoma

Diffuse large B cell lymphoma (DLBCL) is the most common lymphoid malignant tumor, accounting for 30–40% of NHL derived from GC B cells. DLBCL occurs primarily in elderly adults, less frequently in young adults, and rarely in children. Approximately 10–15% of patients are EBV^+^ DLBCL, and this is most prevalent in Asia (31, 32). The large B cells in EBV^+^ DLBCL are clonal centroblastic B cells, which in most cases, express the latency II program (33). Several studies have reported how these EBV^+^ lymphoma cells escape host immune surveillance. Senescence of the immune system related to the aging process that leads to the defective surveillance of EBV may play a role in pathogenesis (33). The dysregulated expression of PD-L1 and PD-L2 caused by a genetic truncation of 3′-UTR has also been identified in EBV^+^ DLBCL, but not in EBV- DLBCL, which provides another explanation of why highly immunogenic EBV^+^ DLBCL can escape immune surveillance in young adults (34).

## Post-transplant Lymphoproliferative Disease

**Figure 1:**
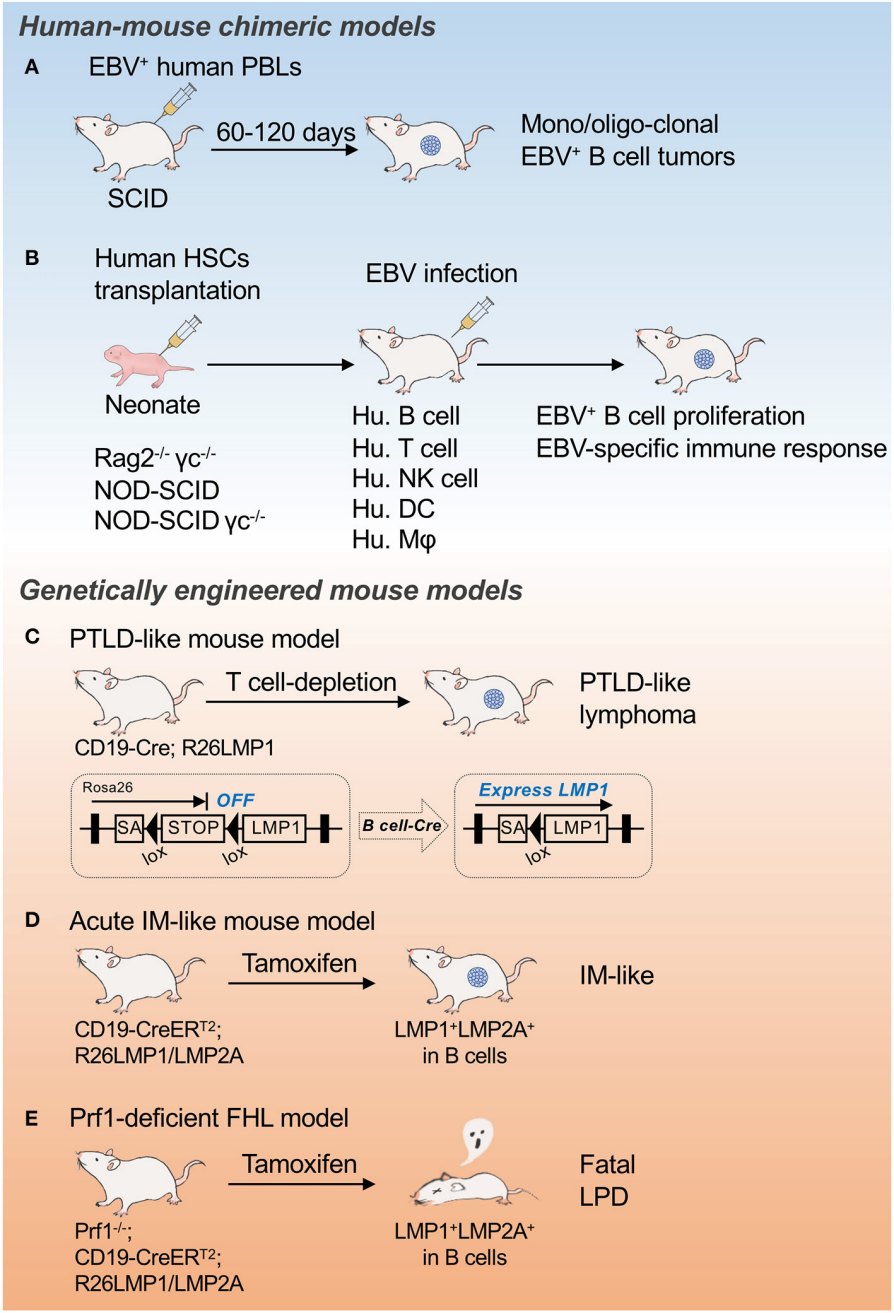
Mouse models for EBV infection and associated malignancies. Schematic illustrations of EBV-seropositive human peripheral blood B cell transfer model **(A)**, Mouse models of EBV infection and associated malignancies **(B)**, PTLD-like mouse model **(C)**, Acute IM-like mouse model **(D)**, and Prf1-deficient FHL model **(E)**. SA, splice acceptor; PBL, peripheral blood lymphocytes; PTLD, post-transplant lymphoproliferative disease; IM, infectious mononucleosis; FHL, familial hemophagocytic lymphohistiocytosis; LPD, lymphoproliferative disease.

Post-transplant lymphoproliferative disease (PTLD) encompasses a heterogenous group of lymphocytic proliferations characterized by the proliferative expansion of lymphocytes, mostly EBV^+^ B cells, in immunocompromised patients due to treatment with immunosuppressive drugs after solid organ or hematopoietic stem cell transplantation. PTLD typically exhibits type III latency features. The World Health Organization (WHO) classification categorizes the disease into IM-like early lesions, polymorphic lymphomas (P-PTLD), and monomorphic lymphomas (M-PTLD). Virtually all early lesions are polyclonal and do not have any known molecular alterations. Many P-PTLDs and most M-PTLDs are clonal. Compared to P-PTLD, M-PTLDs harbor more karyotypic abnormalities. A murine PTLD model expressing LMP1 in B cells in which breaking immune surveillance results in rapid, fatal lymphoproliferation and lymphomagenesis has been established (35) (Figure 1C).

## Acquired Immunodeficiency Syndrome–Related Lymphoma

EBV-positive lymphomas are highly associated with immunocompromised patients who have AIDS caused by human immunodeficiency virus (HIV) infection (Table 1). In AIDS patients, the incidences of DLBCL, HL, BL, primary central nervous system lymphoma (PCNSL), and primary effusion lymphoma (PEL) are increased because of the lack of T cell–mediated immune surveillance, which suggests that CD4^+^ T cells play central roles. EBV-positive rates in these lymphomas are extremely high (90–100% of PCNSL, HL, PEL, and immunoblastic DLBCL; 30–70% of BL and centroblastic DLBCL), indicating the important role of EBV in the development of lymphoma in AIDS patients (8, 36). The cellular origin of those lymphomas are thought to be mostly V gene–mutated GC B cells or post GC B cells.

## EBV-Seropositive Human Peripheral Blood B Cell Transfer Model

Injection of human peripheral blood lymphocytes (PBLs) from EBV-seropositive donors into severe combined immunodeficient (SCID) mice allows the development of EBV^+^ B cell tumors within weeks that resemble the lymphoblastoid cell line (LCL) generated by EBV infection of normal B cells *in vitro* (Figure 1A). The PBL-derived tumors resembling EBV^+^ large cell lymphoma in immunosuppressed patients formed monoclonal or oligoclonal foci (37). The remarkable efficiency of clonal tumor development in the human PBL-SCID model suggests that lymphomagenesis involves the direct outgrowth of EBV-transformed B cells without the requirement of secondary genetic alterations. This transfer model is unable to induce a host immune response to EBV^+^ B cells, however, which should happen in lymphoproliferative diseases or lymphomas.

## Mouse Models of EBV Infection and Associated Malignancies

Human beings are the only natural host of EBV. As only New World monkeys can be infected by EBV experimentally, there is a major limitation to using primates as an animal model of EBV-associated pathogenesis. Mice reconstituted with human immune cells, called humanized mice, have been developed to address the pathology of human hematopoietic cells, including EBV infection to human B cells (Figure 1B). Transplantation of human HSCs into severely immunocompromised mice such as Rag2^−/−^ γc^−/−^, NOD-SCID, and NOD-SCID γc^−/−^ mice allows the reconstitution of a functional human immune system, including B cells, T cells, natural killer (NK) cells, dendritic cells (DCs), and macrophages. The administration of live EBV to those reconstituted mice successfully infected the reconstituted human B cells, developed LMP1^+^ B cell proliferation, and mounted human MHC class I– and class II–restricted adaptive immune responses to EBV infection (38–40). Although this system has critical problems in human T cell selection on a mouse thymic background and the T cells generated a discriminated self from allogeneic MHC, this approach provides a tool with which to study pathogens that specifically target the human immune system and to test potential therapeutic interventions.

## Genetically Engineered EBV Models

The reconstitution of EBV pathogenesis as well as lymphomagenesis through the conditional and timed expression of limited EBV molecules in mice without virus infection is challenging. We previously generated mice expressing LMP1 specifically in B cells in early development (CD19-Cre; R26LMP1 mouse, PTLD-like lymphoma model) (Figure 1C). Similar to EBV-infected human B cells, LMP1^+^ mouse B cells were efficiently eliminated by T cells whereas disrupting immune surveillance resulted in rapid, fatal lymphoproliferation and lymphomagenesis (35). These results indicate the central role of LMP1 in the surveillance and transformation of EBV-infected B cells *in vivo*, and resulted in the establishment of the first preclinical mouse model for immune suppression-dependent lymphomagenesis.

The acute EBV infection of naïve B cells in mice can be modeled through the timed expression of LMP1 and LMP2A by tamoxifen-mediated Cre recombination (CD19-CreERT2; R26LMP1/LMP2A mouse) (Figure 1D). Although lethal when induced in all B cells, the induction of LMP1 and LMP2A in just a few naïve B cells initiated a phase of rapid B cell expansion followed by a proliferative T cell response that cleared the LMP-expressing B cells. Interfering with T cell activity prevented the clearance of LMP-expressing B cells (41, 42). Using this system, primary human immunodeficiency diseases can be reconstructed, such as perforin (Prf1)-deficient FHL (Figure 1E), which causes a life-threatening EBV-related immunoproliferative syndrome in humans (42). Thus, the timed expression of LMP1 together with LMP2A in subsets of mouse B cells allows the study of the major clinically relevant features of human EBV infection *in vivo*, thereby providing a unique animal model that may be useful for therapeutic testing.

As described above, although EBV infection in humanized mice has been successfully used to recapitulate virally driven B cell lymphomagenesis, this approach completely lacks the hematopoietic cell environment that should be generated normally (43). As such, it remains meaningful to study lymphomagenesis in mouse models that induce EBV-driven lymphoma in an *in vivo* environment that contains naturally occurring T cells after education and selection, and that also carries a normal set of innate and acquired immune cells with a uniform genetic background. Furthermore, mouse cytokines and chemokines cannot fully support human hematopoiesis and *in vivo* dynamics of human immune cells. A genetic engineering approach has been used to attempt an immunocompromised latency III-like lymphoma model in which EBNA3A, LMP1, and LMP2A are simultaneously expressed in GC B cells. In immunocompetent mice, B cells expressing EBV genes are efficiently eliminated by T cells; however, in immunocompromised recipient mice, tumors similar to human EBV^+^ lymphomas were formed. EBNA3A or a recurrent activating mutation of the B cell transcription factor EBF1, a functional proxy of EBNA3A, interplays with other EBV oncogenes in B cell transformation (19).

## Conclusion

Genetic mouse models have shown that the expression of LMP1 is sufficient to model the major features of EBV infection in mice, namely, immunogenicity and tumorigenesis, despite the fact that EBV is endemic to humans. This is of particular interest because the T cell–mediated immune surveillance of LMP1^+^ B cells stands in contrast to the belief that the human immune system has evolved to prevent the expansion of EBV-infected B cells. The virus might have evolved to be recognized by the mammalian immune system, likely because lifelong latent infection is advantageous over fatal infection. The development of preclinical mouse models for a range of EBV-associated pathologies is challenging but may open the path to the development of new therapeutic approaches.

## Author Contributions

SH wrote the manuscript and prepared the figure and table. TY conceptualized and wrote the manuscript and edited the figure and table. Both authors contributed to the article and approved the submitted version.

## Conflict of Interest

The authors declare that the research was conducted in the absence of any commercial or financial relationships that could be construed as a potential conflict of interest.
